# The Role of *AKR1B1* in Diabetic Nephropathy: A Genetic Association Study and Meta-Analysis

**DOI:** 10.3390/genes17060652

**Published:** 2026-05-31

**Authors:** Maria Tziastoudi, Christos Cholevas, Efthimios Dardiotis, Evangelia E. Tsironi, Maria Divani, Theodoros Eleftheriadis, Ioannis Stefanidis

**Affiliations:** 1 Department of Nephrology, Faculty of Medicine, University of Thessaly, 41110 Larissa, Greece; mariadivani@yahoo.com (M.D.); teleftheriadis@uth.gr (T.E.); stefanid@uth.gr (I.S.); 2 Laboratory of Pharmaceutical Technology, Division of Pharmaceutical Technology, School of Pharmacy, Faculty of Health Sciences, Aristotle University of Thessaloniki, 54124 Thessaloniki, Greece; ccholevas@auth.gr; 3 Department of Neurology, University Hospital of Larissa, Faculty of Medicine, School of Health Sciences, University of Thessaly, 41110 Larissa, Greece; edar@uth.gr; 4 Department of Ophthalmology, Faculty of Medicine, School of Health Sciences, University of Thessaly, 41110 Larissa, Greece; e_tsironi@hotmail.com

**Keywords:** diabetes mellitus, diabetic nephropathy, aldo-keto reductase family 1 member B (*AKR1B1*), genetic association study, meta-analysis

## Abstract

Background: Diabetic nephropathy (DN) is a major microvascular complication of diabetes mellitus with a strong genetic component. The aldo-keto reductase family 1 member B *(AKR1B1)* gene has been implicated in hyperglycemia-induced renal damage. This study aimed to investigate the association between *AKR1B1* variants and DN through a genetic association study (GAS) and a comprehensive meta-analysis. Methods: A case–control study was conducted in a Greek population, including 190 DN patients, 150 T2DM patients without nephropathy, and 238 healthy controls. Five tagging single-nucleotide polymorphisms (SNPs) in *AKR1B1* were genotyped. Associations were assessed using the generalized odds ratio (OR_G_). A systematic literature search was performed, and eligible studies were included in a meta-analysis using a random-effects model. Results: In the case–control study, none of the examined SNPs showed a significant association with DN in any comparison model. However, meta-analysis results demonstrated a significant association for the promoter variant −106 C>T (rs759853) in diabetic individuals without nephropathy versus DN cases (OR_G_ = 1.389, 95% CI: 1.134–1.700). The (CA)n polymorphism showed a non-significant overall effect but was associated with increased risk in subgroups of Asians (OR_G_ = 1.455, 95% CI: 1.042–2.032) and T2DM patients (OR_G_ = 1.357, 95% CI: 1.039–1.771). No associations were observed when healthy controls were used. Conclusions: While no significant associations were detected in the single-cohort analysis, meta-analytic evidence supports a role of regulatory *AKR1B1* variants in DN susceptibility. These findings highlight the importance of control selection and suggest that *AKR1B1* may influence progression to nephropathy rather than diabetes risk per se.

## 1. Introduction

Diabetic nephropathy (DN) is characterized by a persistent deterioration of kidney function, indicated by an estimated glomerular filtration rate (eGFR) < 60 mL/min/1.73 m^2^ for at least three months, and may occur with or without albuminuria, defined as urinary albumin loss greater than 300 mg per day [1,2]. DN is the most common underlying cause of end-stage renal disease (ESRD) globally and is associated with a substantially increased risk of long-term mortality among individuals with diabetes and renal impairment [3]. Its onset and progression result from a combination of pathological mechanisms, including abnormal glomerular haemodynamics, injury to podocytes, and ongoing tubulointerstitial inflammation leading to fibrosis [4,5]. Present treatment strategies focus on tight control of blood pressure and blood glucose levels by incorporating into the therapeutic armamentarium agents like renin–angiotensin system (RAS) blockers and non-steroidal mineralocorticoid receptor antagonists (MRAs), as well as more recently introduced therapies such as sodium–glucose cotransporter 2 (SGLT2) inhibitors and glucagon-like peptide 1 receptor agonists (GLP1RAs) [6,7,8,9].

Chronic hyperglycaemia initiates a series of pathogenic cellular responses and signaling pathways within the kidney. When intracellular glucose levels exceed the capacity of glycolysis, metabolic flux is redirected into four principal pathways: the polyol pathway, advanced glycation end-product (AGE) formation, protein kinase C (PKC) activation, and the hexosamine biosynthetic pathway [4,10]. Activation of these pathways intensifies oxidative stress, disrupts redox homeostasis, and modifies gene expression, thereby establishing a self-perpetuating cycle of cellular damage [11,12].

Even patients who maintain optimal glycemic control can still develop nephropathy, suggesting that factors beyond blood glucose—such as genetic susceptibility and epigenetic regulation—play an important role [13,14,15]. In addition, the well-documented familial aggregation of diabetic nephropathy further supports the involvement of inherited predisposition in its pathogenesis across both types of diabetes mellitus [16,17]. Nevertheless, the specific genetic determinants underlying this susceptibility remain largely undefined despite extensive efforts [18,19,20,21,22,23,24].

*AKR1B1* encodes aldose reductase, a cytosolic nicotinamide adenine dinucleotide phosphate (NADPH)-dependent enzyme that catalyzes the first and rate-limiting step of the polyol pathway, reducing glucose to sorbitol [25,26,27,28]. Under hyperglycemic conditions, increased AKR1B1 activity enhances sorbitol accumulation and markedly consumes NADPH. This leads to osmotic stress and depletion of reducing equivalents. As a result, glutathione regeneration is impaired, and oxidative stress is amplified [29]. In parallel, AKR1B1 reduces a wide range of reactive aldehydes generated during lipid peroxidation, contributing to cellular detoxification and redox control. However, this protective function also relies on NADPH, further linking AKR1B1 activity to redox imbalance [30]. Thus, AKR1B1 occupies a central biochemical position connecting the polyol pathway, oxidative stress, and cellular defense mechanisms.

*AKR1B1* is expressed in several tissues, including the kidney [31]. Overall, *AKR1B1* expression is closely linked to the pathogenesis of diabetic microvascular complications, as its increased activity promotes sorbitol accumulation and subsequent cellular damage [32].

In an effort to elucidate the contribution of the *AKR1B1* gene in the course of DN, we performed a genetic association study (GAS) in a Greek cohort genotyping five variants across the *AKR1B1* gene. In order to provide the most informed evaluation about the role of this gene in DN, we also conducted a systematic review and meta-analysis of all available GAS that examine the association of genetic variants across *AKR1B1* with DN.

## 2. Materials and Methods

### 2.1. Association Study

Detailed descriptions of the study methodology and the demographic profile of the participants have been published elsewhere [33]. In summary, the cohort comprised 190 individuals diagnosed with diabetic nephropathy (DN), 150 patients with type 2 diabetes mellitus (T2DM) without evidence of microvascular complications, and 238 non-diabetic healthy controls. All participants were assessed at the Ophthalmology and Nephrology Departments of the University Hospital of Larissa, Greece.

Diabetic nephropathy was defined by the presence of sustained macroalbuminuria, characterized by urinary albumin excretion exceeding 300 mg per 24 h (>200 μg/min), regardless of serum creatinine concentrations. Ethical approval for the study was granted by the Ethics Committee of the University of Thessaly (3/17-03-2014), and all participants provided written informed consent prior to inclusion.

Genotyping procedures were carried out as described previously [33]. In total, five tagging single-nucleotide polymorphisms (SNPs) within the *AKR1B1* gene were analyzed, including rs2259458, rs2734653, rs2670230, rs1790998 and rs17188118. Tag SNP selection was based on a linkage disequilibrium threshold of r^2^ ≥ 0.8 and a minor allele frequency (MAF) exceeding 0.05. To verify genotyping reliability, a minimum of 10% of samples were randomly chosen and re-genotyped as part of internal quality control procedures. All genotyping assays were performed by personnel blinded to the clinical characteristics of the study participants.

Associations between genotype distributions and disease susceptibility were evaluated using the generalized odds ratio (OR_G_) approach [34,35]. Genotype frequencies among healthy control subjects were additionally examined for deviations from the Hardy–Weinberg equilibrium (HWE). OR_G_ calculations were conducted using the ORGGASMA software package (http://biomath.med.uth.gr; accessed on 15 January 2026). All statistical analyses were carried out with SPSS software, version 29.0 for Windows (SPSS Inc., Chicago, IL, USA).

### 2.2. Meta-Analysis

A literature search was conducted in PubMed using combinations of the terms [(“*AKR1B1*” or aldo-keto reductase) and (“diabetic nephropathy” or “diabetic kidney disease”)] (last accessed on 19 December 2025). The abstracts of the retrieved records were reviewed to determine study relevance. In addition, relevant publications were identified through the National Human Genome Research Institute (NHGRI) Catalog of Published Genome-Wide Association Studies (http://www.genome.gov/gwastudies/, accessed on 30 January 2026) by applying the disease keyword “diabetic nephropathy.” Reference lists of eligible articles, as well as previously published meta-analyses, were also examined to identify further studies. No attempts were made to obtain unpublished datasets from study authors, and only articles available in the English language were included.

Studies were considered eligible if they satisfied the following conditions: (i) inclusion of individuals presenting with sustained microalbuminuria or macroalbuminuria, irrespective of the presence of diabetic retinopathy; (ii) inclusion of comparison groups consisting of diabetic individuals with normoalbuminuria or preserved renal function and/or non-diabetic healthy participants; (iii) availability of complete genetic information, reported as genotype distributions or allele frequencies, with studies presenting only combined genotype results being excluded; and (iv) enrollment of human participants. Both type 1 diabetes mellitus (T1DM) and type 2 diabetes mellitus (T2DM) populations were acceptable. Studies that focused exclusively on cases without persistent microalbuminuria were not included.

Studies addressing disease progression, severity, phenotypic modulation, therapeutic response, or survival outcomes were not considered. In addition, case reports, editorials, review articles, publications in languages other than English, and studies employing alternative designs—such as family-based analyses—were excluded. Study selection was carried out independently by two reviewers (M.T. and I.S.); their assessments were subsequently compared, and any discrepancies were resolved through mutual agreement.

From each eligible publication, we collected data on the first author, publication year, ethnic background, PubMed identification number, diabetes type, country of origin, and reported phenotype. For both case and control groups, information was gathered on sample size, duration of diabetes, inclusion criteria, and the application of matching criteria. When available, complete genotype distributions or allele frequency data were also extracted for genetic analyses.

The relationship between genotype distributions and diabetic nephropathy (DN) was evaluated using the generalized linear odds ratio (OR_G_) [34,35]. This measure quantifies the likelihood that an individual with a greater mutational burden is affected by the disease compared with the likelihood of being unaffected. More specifically, the OR_G_ reflects the ratio of case–control pairs in which the case carries a higher mutational load to those in which the control carries a higher mutational load. In this way, the OR_G_ provides an indication of whether the mutational burden associated with a given variant contributes to disease susceptibility.

The generalized odds ratio (OR_G_) was computed for each genetic variant for which complete genotype data were available. When only allele frequency information was reported, analyses were conducted under an allele contrast model. Variants were eligible for meta-analysis provided that data from at least two independent studies were available. Summary odds ratios were calculated using the random-effects approach proposed by DerSimonian and Laird [36]. Results are reported as odds ratios—generalized for genotype-based analyses or pooled for allele-based analyses—along with their corresponding 95% confidence intervals (CIs). Inter-study heterogeneity was evaluated using Cochran’s Q test, with statistical significance defined at *p* < 0.10, and its magnitude was quantified using the *I*^2^ statistic [37,38]. This measure ranges from 0% to 100% and is not influenced by the number of studies, with higher values indicating greater heterogeneity. The calculation of OR_G_ was performed using dedicated software designed for generalized odds ratio analyses in genetic association studies and meta-analyses (ORGGASMA; http://biomath.med.uth.gr, accessed on 15 February 2026).

For each individual study, the Hardy–Weinberg equilibrium (HWE) in control groups was assessed using Fisher’s exact test when genotype data were available. In cases where only allele counts were reported, the evaluation of HWE deviations was based on the original authors’ conclusions. Finally, potential small-study effects were investigated using Egger’s regression test [39].

## 3. Results

### 3.1. Association Study

The study population included 190 DN cases, 150 diseased controls and 238 healthy controls. All participants were Caucasians of Greek origin. Demographic data and clinical profile are summarized in Table 1, as have also been described elsewhere [33]. Five SNPs in the *AKR1B1* gene (rs2259458, rs2734653, rs2670230, rs1790998, and rs17188118) were successfully genotyped and analyzed across three groups.

The genotype distributions of the five variants (rs2259458, rs2734653, rs2670230, rs1790998, and rs17188118) and the respective OR_G_ are shown in Table 2, Table 3 and Table 4. Overall, genotype distributions were comparable across groups, and no variant reached statistical significance in any comparison.

### 3.2. Meta-Analysis

**Figure 1:**
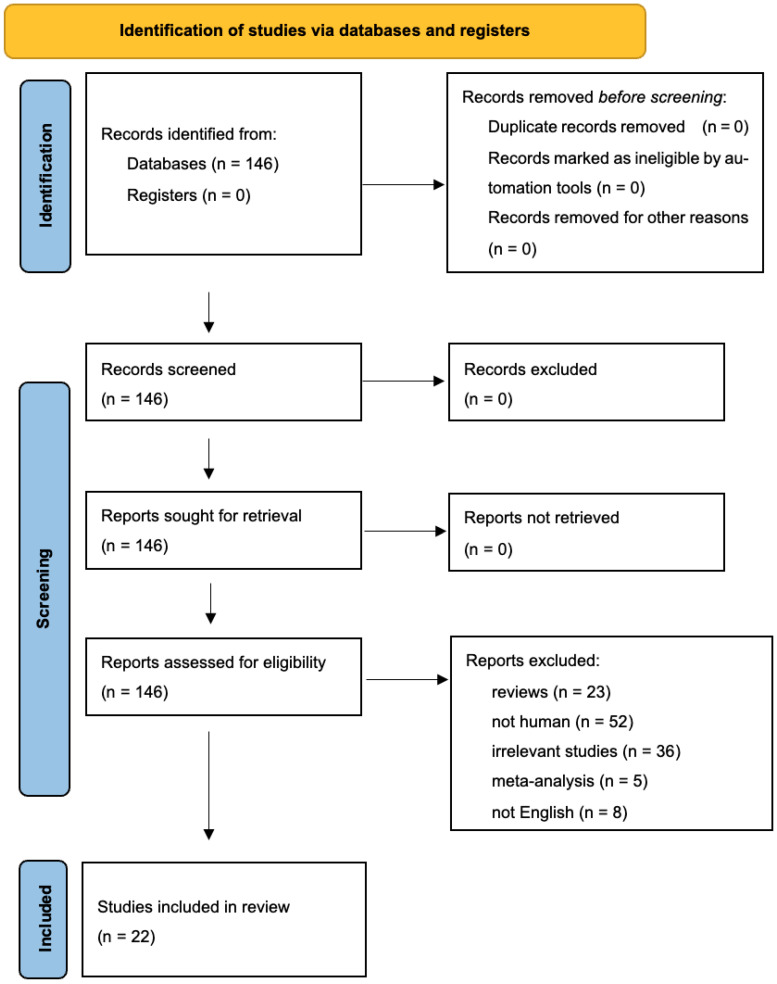
Flowchart showing how studies were selected for meta-analysis.

The literature search yielded 146 PubMed records that met the inclusion criteria. When a study reported data from different populations, each population was considered as a separate dataset. A flowchart summarizing the selection process and reasons for exclusion is provided in Figure 1, while the characteristics of the included studies are detailed in Table 5.

**Figure 2:**
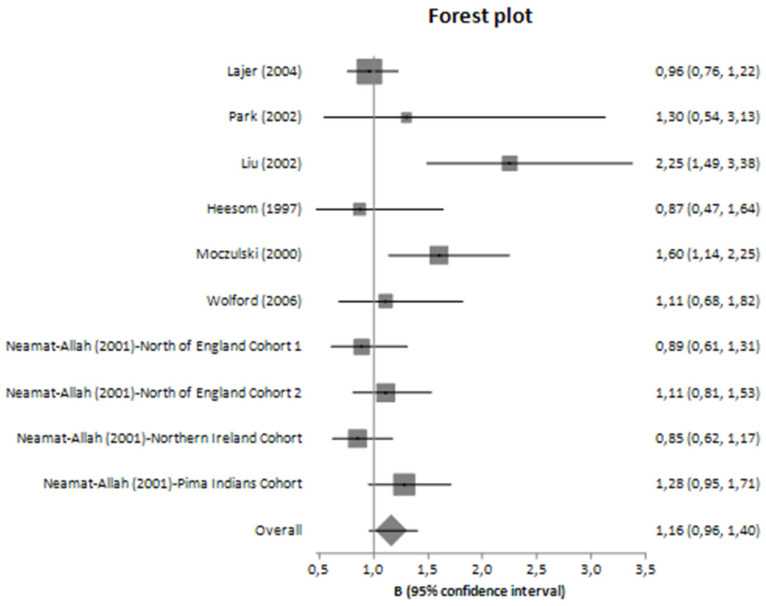
Forest plot of (CA)n polymorphism between diseased controls (diabetic individuals without DN) versus cases (diabetic individuals with DN).

**Figure 3:**
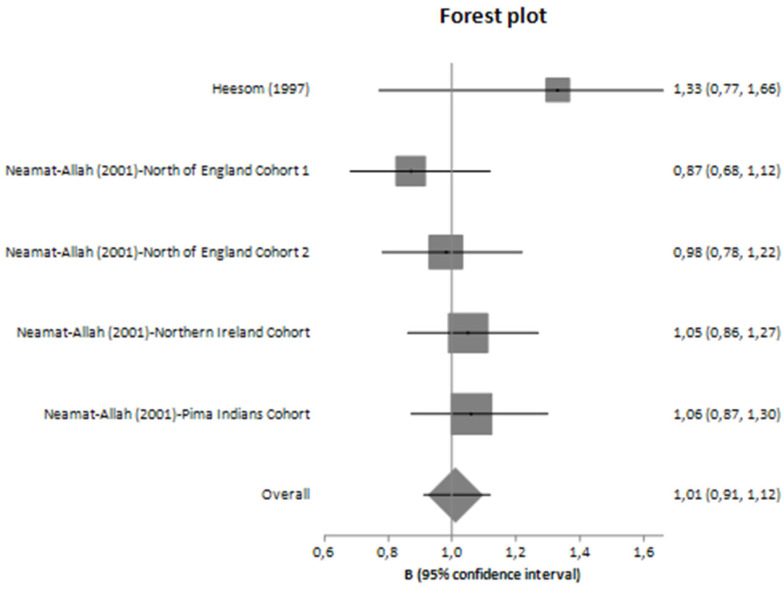
Forest plot of (CA)n polymorphism between healthy controls versus diseased controls (diabetic individuals without DN) versus cases (diabetic individuals with DN).

**Figure 4:**
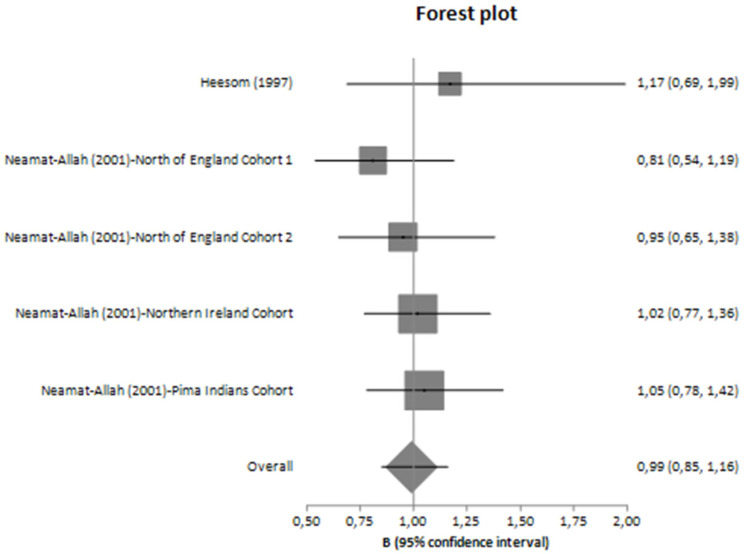
Forest plot of (CA)n polymorphism between healthy controls versus cases (diabetic individuals with DN).

**Figure 5:**
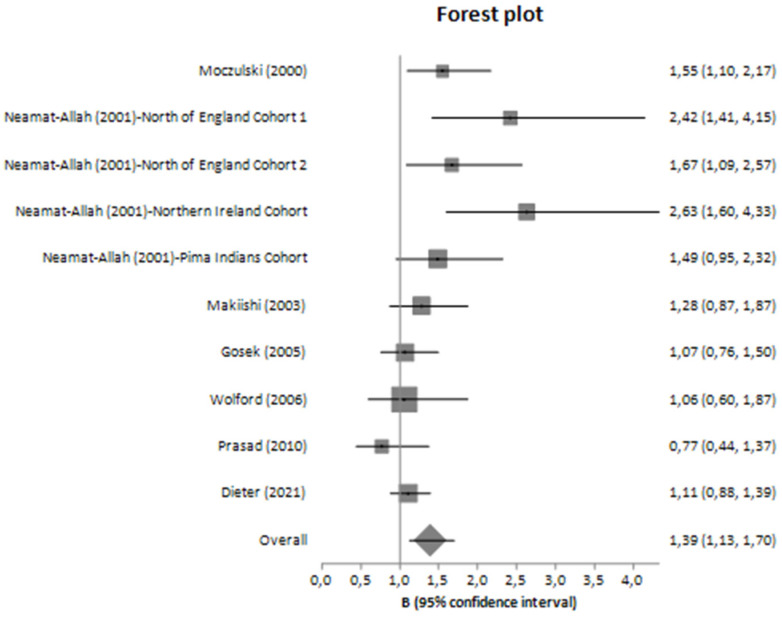
Forest plot of −106 C/T polymorphism between diseased controls (diabetic individuals without DN) versus cases (diabetic individuals with DN).

**Figure 6:**
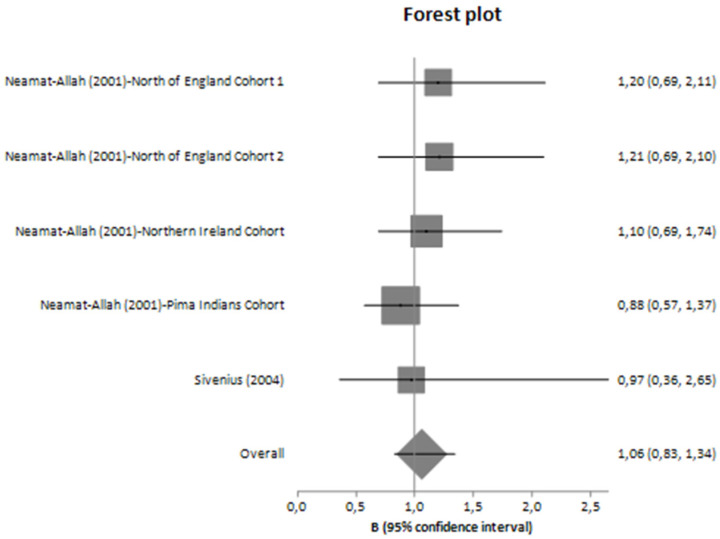
Forest plot of −106 C/T polymorphism between healthy controls versus cases (diabetic individuals with DN).

**Figure 7:**
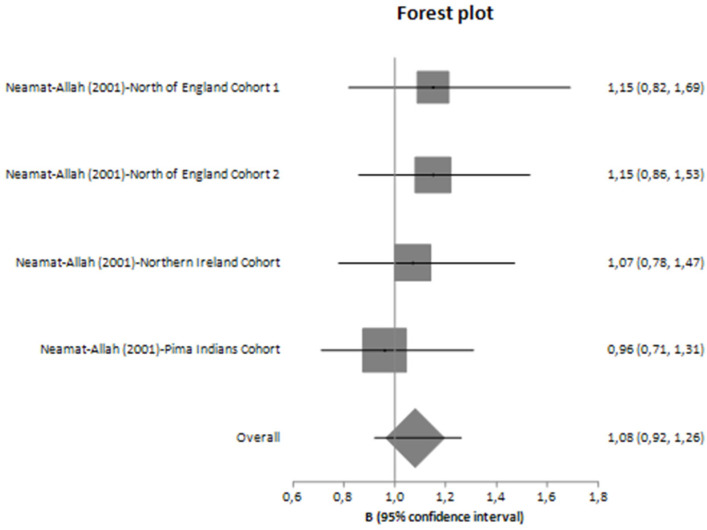
Forest plot of −106 C/T polymorphism between healthy controls versus diseased controls (diabetic individuals without nephropathy) versus cases (diabetic individuals with DN).

A meta-analysis was conducted for the (CA)n microsatellite polymorphism and the −106 C>T variant (rs759853), as well as selected SNPs based on available genotype or allele data. The studies were published between 1997 and 2021. The forest plots are presented in Figure 2, Figure 3, Figure 4, Figure 5, Figure 6 and Figure 7. Meta-analysis results are presented in Table 6 and Table 7.

### 3.3. Genotype-Based Meta-Analysis

For the (CA)n polymorphism in studies using diseased controls, the pooled estimate indicated a non-significant trend toward increased risk (OR_G_ = 1.162, 95% CI: 0.962–1.404), accompanied by moderate heterogeneity (I^2^ = 60.0%). Stratified analyses showed statistically significant associations in Asians (OR_G_ = 1.455, 95% CI: 1.042–2.032) and in type 2 diabetes mellitus (T2DM) (OR_G_ = 1.357, 95% CI: 1.039–1.771), whereas no significant associations were observed in Caucasians (OR_G_ = 1.035, 95% CI: 0.857–1.251), type 1 diabetes mellitus (T1DM) (OR_G_ = 1.018, 95% CI: 0.806–1.286), or studies conforming to the Hardy–Weinberg equilibrium (OR_G_ = 1.397, 95% CI: 0.992–1.968).

In contrast, analyses restricted to healthy controls demonstrated no evidence of association (OR_G_ = 0.991, 95% CI: 0.847–1.159) and no between-study heterogeneity (I^2^ = 0%). Consistent null findings were observed across all subgroups, including Caucasians, Asians, T1DM, and T2DM.

Similarly, when both healthy and diseased controls were combined, the overall effect remained non-significant (OR_G_ = 1.010, 95% CI: 0.911–1.121) with no heterogeneity (I^2^ = 0%), and subgroup analyses yielded uniformly null results.

For rs759853 (−106 C>T) variant in the comparison of diseased controls versus cases, the OR_G_ was 1.389 (95% CI: 1.134–1.700), indicating a statistically significant association. Moderate heterogeneity was observed (I^2^ = 59.5%, PQ = 0.008).

In contrast, no significant association was detected for rs759853 when comparing healthy controls versus cases (OR_G_ = 1.057; 95% CI: 0.831–1.344; *I*^2^ = 0%) or in the combined healthy controls versus diseased controls versus cases analysis (OR_G_ = 1.078; 95% CI: 0.922–1.260; *I*^2^ = 0%).

### 3.4. Allele-Based Meta-Analysis

In allele-based analyses for diseased controls versus cases, the (CA)n polymorphism (8 studies) yielded a pooled OR of 1.154 (95% CI: 0.910–1.464), indicating no significant association. Similarly, rs2361634 (3 studies; OR = 0.788; 95% CI: 0.552–1.125), rs5918764 (3 studies; OR = 1.076; 95% CI: 0.617–1.876), and ss95212308 (3 studies; OR = 1.041; 95% CI: 0.834–1.300) were not significantly associated with disease risk.

## 4. Discussion

In the present study, we investigated the association between five *AKR1B1* polymorphisms (rs2259458, rs2734653, rs2670230, rs1790998, rs17188118) and DN in a case–control study design including healthy controls, diabetic controls without nephropathy, and diabetic individuals with diabetic nephropathy. None of the analyzed variants demonstrated a statistically significant association under any of the examined comparisons (HT vs. DN, DC vs. DN, or overall). However, our meta-analysis identified a significant association for the promoter variant −106 C>T (rs759853), as well as for the (CA)n polymorphism in the comparison of diseased controls versus cases, supporting a potential role of *AKR1B1* in DN susceptibility. The (CA)n polymorphism was revealed statistically significant in subgroup analyses of Asians and T2DM subjects, whereas the promoter variant −106 C>T was statistically significant in all analyses, primary and subgroup, except in the subgroup analysis of Asians.

Despite the strong biological plausibility of *AKR1B1* as a candidate gene for DN, our single-cohort results did not reveal significant associations for the five intragenic SNPs examined. Aldose reductase, encoded by *AKR1B1*, is the rate-limiting enzyme of the polyol pathway, which is upregulated under hyperglycemic conditions and contributes to osmotic stress, oxidative imbalance, and advanced glycation end-product formation in renal tissues [32]. Therefore, genetic variants affecting the expression or activity of aldose reductase could modulate individual susceptibility to microvascular complications.

The lack of association in our population may reflect modest effect sizes, limited statistical power for rare genotypes (particularly rs17188118), or population-specific linkage disequilibrium patterns. It is also possible that regulatory polymorphisms exert stronger functional consequences than coding or intronic variants, consistent with our meta-analytic findings for the promoter SNP−106 C>T (rs759853).

Regarding the contribution of (CA)n polymorphism in DN, the results are not consistent. More specifically, our main meta-analysis between diseased controls versus cases did not reveal a statistically significant contribution of this variant. Similarly, non-significant results were reported in sub-analyses of Caucasians and T1DM. However, significant results were reported in sub-analyses of Asians and T2DM.

The (CA)n dinucleotide repeat located in the 5′ regulatory region of *AKR1B1* has been widely investigated in both Caucasian and Asian populations, as well as in cohorts with T1DM and T2DM. Several studies conducted in Caucasian populations have reported that the Z−2 allele is associated with an increased risk of DN [40,43,44,53]. In contrast, other investigations in Caucasian cohorts have not identified a significant association between this polymorphism and DN susceptibility [46,54,56,57,58,61].

With regard to studies conducted in Asian populations, only one investigation reported a significant association between the (CA)n polymorphism and DN [42]. All other studies in Asian cohorts failed to demonstrate a significant relationship between this variant and DN risk [41,45,46,52,57].

Regarding sub-analyses according to diabetes type, findings in T1DM have been inconsistent. While several studies reported significant associations [40,43,44,53], other investigations failed to demonstrate statistically significant results [46,54,55,58]. In T2DM, only one study reported a significant association [42]. All other studies failed to demonstrate a statistically significant relationship between this variant and DN susceptibility [41,45,52,56,57].

With regard to the −106 C>T polymorphism, both the overall analysis and the subgroup analyses in Caucasians, T1DM, and T2DM yielded statistically significant results suggesting that the T allele may increase susceptibility among diabetic individuals. This is biologically plausible, as promoter variants can alter transcription factor binding and modulate gene expression. Increased aldose reductase expression could amplify polyol pathway flux under hyperglycemia, exacerbating glomerular and tubular injury. However, the subgroup analysis in Asian populations did not demonstrate a statistically significant association.

In Caucasian populations, only one study did not report a statistically significant association [45]. In contrast, the remaining studies identified a significant association between this polymorphism and DN [44,46,47,48], confirming our significant meta-analysis result. In Asian populations, the non-significant result of our meta-analysis is consistent with the negative findings reported in previously published studies [46,52,57].

In both subgroup analyses, T1DM and T2DM, the meta-analysis yielded statistically significant results. More specifically, in the T1DM subgroup analysis, two studies reported significant associations [46,53], whereas three studies did not demonstrate statistically significant results [54,55,58]. In the T2DM subgroup analysis, the findings were inconsistent. One study reported a significant association [46], whereas the remaining three studies did not identify statistically significant results [52,56,57].

Interestingly, no significant association was observed when healthy controls were included in the comparisons. This pattern suggests that the variants −106 C>T (rs759853), as well as the (CA)n polymorphism, may influence the development of DN when diabetes mellitus is present rather than the risk of developing diabetes per se, emphasizing the importance of appropriate control selection in genetic studies of complications. Therefore, studies using only healthy controls may dilute associations specific to microvascular complications.

Three additional SNPs (rs2361634, rs5918764, and ss95212308) were examined in large T1DM cohorts from Denmark, Finland, and France [61]. Our allele-based meta-analysis did not identify significant associations for these variants.

A key strength of our work is the combined approach, integrating a well-characterized case–control study with a comprehensive meta-analysis that incorporates multiple cohorts, thereby increasing statistical power and improving the precision of risk estimates compared with individual studies. In addition, subgroup analyses by ethnicity and diabetes type allowed exploration of potential sources of heterogeneity.

However, several limitations should be acknowledged. First, heterogeneity across studies—which may reflect differences in study design, population characteristics, or allele classification methods—limits the precision of pooled estimates. For instance, an unequal number of cases and controls that is present in some studies, which, although common in case–control designs, may reduce statistical efficiency and affect the precision of effect estimates without introducing systematic bias. Second, differences in nephropathy definitions and control selection criteria may introduce bias [62]. Third, functional validation of the identified associations was beyond the scope of this study and remains necessary to clarify the mechanistic impact of promoter variation. Furthermore, the number of studies available for certain subgroup analyses, particularly those involving T1DM, was relatively small. In addition, our analysis was based on aggregated data rather than individual-level data, which prevented evaluation of gene–gene and gene–environment interactions. Finally, environmental and clinical factors such as glycemic control, duration of diabetes, and treatment regimens could not be uniformly controlled across studies.

Our single-case–control data do not support a major role for the examined intragenic *AKR1B1* SNPs in DN susceptibility. However, the present meta-analysis provides evidence that the promoter variant −106 C>T (rs759853) and (CA)n polymorphism may contribute to nephropathy risk among diabetic individuals. These findings reinforce the biological relevance of aldose reductase in diabetic microvascular complications and highlight the potential importance of regulatory genetic variation.

Future studies should focus on large, multiethnic prospective cohorts with standardized nephropathy definitions, fine-mapping and haplotype analyses across the *AKR1B1* locus, functional assays to determine the impact of −106 C>T (rs759853) variant and (CA)n alleles on transcriptional activity, as well as integration of genetic data with transcriptomic and epigenetic profiling in renal tissue. Such approaches will help clarify whether *AKR1B1* variation plays a clinically meaningful role in DN pathogenesis and whether it may inform risk stratification or therapeutic targeting of the polyol pathway.

These findings indicate that DN is a multifactorial disorder characterized by complex epistatic effects and gene–environment interactions. Consequently, relying on a single genetic approach, such as candidate gene association studies, is unlikely to yield definitive conclusions [63,64]. In contrast, hypothesis-free approaches like genome-wide association studies (GWAS) offer a more comprehensive framework for elucidating the genetic contribution to DN [65,66]. Although GWAS provide a more powerful strategy for addressing genetic complexity, findings from candidate gene studies remain valuable, as they can support the replication of existing associations and help identify true genetic effects that warrant prioritization in future research.

## 5. Conclusions

In the present case–control study, none of the investigated *AKR1B1* SNPs demonstrated a significant association with diabetic nephropathy under any comparison model. However, meta-analytic evidence supports a significant association between the −106 C>T (rs759853) promoter variant, as well as (CA)n polymorphism, and nephropathy risk among diabetic individuals, particularly in comparisons between diseased controls and cases. These findings should be interpreted with caution due to substantial heterogeneity across studies, limited sample size, and the absence of functional or patient-level data. Therefore, further large-scale and mechanistic studies are warranted to confirm these observations.

## Figures and Tables

**Table 1:** Demographic data and clinical profile of the study population.

Parameters	Case–Control Study Population Groups (*n* = 578)					
	HC	DM	*p*-Value	DM-DN	DM + DN	*p*-Value
N	238	340	n.a.	150	190	n.a.
Gender [m; n (%)]	136 (42.9)	181 (57.1)	0.361	74 (47.7)	107 (54.3)	0.305
Age (years)	71 ± 9.2	68 ± 8.9	<0.001	68 ± 9.1	69 ± 8.8	0.380
DM duration (years)	n.a.	16.3 ± 8.0	n.a.	15.7 ± 8.3	16.8 ± 7.8	0.203
HbA1c	n.d.	7.36 ± 1.32	n.a.	7.20 ± 1.34	7.47 ± 1.29	0.064
Insulin treatment (%)	n.d.	105 (32.3)	n.a.	50 (32.3)	55 (27.9)	0.412
Hypertension (%)	0	222 (63.4)	<0.001	97 (63.0)	125 (63.8)	0.912
Cardiovascular disease (%)	0	110 (31.3)	<0.001	41 (26.5)	69 (35.0)	0.105
Creatinine (mg/dL)	0.77 ± 0.15	1.46 ± 1.37	<0.001	0.90 ± 0.18	1.85 ± 1.67	<0.001
Urea (mg/dL)	30 ± 7.9	59 ± 34	<0.001	42 ± 13.6	71 ± 38.3	<0.001
Albuminuria (mg/d)	36.7 ± 63.5	470 ± 856	0.382	43.9 ± 53.4	783 ± 1020	<0.001
Proteinuria (mg/d)	136.6 ± 118.5	788 ± 1468	0.444	105 ± 80.0	1311 ± 1784	<0.001

HC: healthy controls, DM: diabetes mellitus, DM-DN: diabetic individuals without nephropathy, and DM + DN: diabetic individuals with nephropathy, n.a.: not applicable, n.d: not defined. *p*-values were calculated by the Mann–Whitney U test for continuous variables or the *χ*^2^ test for categorical variables as appropriate. The reported *p*-values reflect unadjusted analyses.

**Table 2:** Results of association analysis between AKR1B1 variants and T2DM-nephropathy in healthy controls versus diseased controls versus cases.

AKR1B1	Alleles	HT	DC	DN	OR_G_
rs2259458	TT	43	26	28	0.97 (0.78, 1.21)
	TG	106	60	101	
	GG	90	66	63	
rs2734653	GG	138	96	102	1.05 (0.82, 1.35)
	GA	85	42	81	
	AA	16	12	7	
rs2670230	CC	88	46	51	1.17 (0.93, 1.46)
	CA	104	71	109	
	AA	47	33	33	
rs1790998	CC	93	57	80	0.96 (0.76, 1.21)
	CA	105	70	75	
	AA	45	23	38	
rs17188118	AA	206	136	170	0.94 (0.60, 1.49)
	AC	26	11	19	
	CC	1	1	2	

HT: healthy controls, DC: diabetic individuals without nephropathy, and DN: diabetic individuals with nephropathy.

**Table 3:** Results of association analysis between AKR1B1 variants and T2DM-nephropathy in diseased controls versus cases.

AKR1B1	Alleles	DC	DN	OR_G_
rs2259458	TT	26	28	0.79 (0.55, 1.14)
	TG	60	101	
	GG	66	63	
rs2734653	GG	96	102	1.35 (0.89, 2.04)
	GA	42	81	
	AA	12	7	
rs2670230	CC	46	51	1.00 (0.69, 1.44)
	CA	71	109	
	AA	33	33	
rs1790998	CC	57	80	0.99 (0.69, 1.42)
	CA	70	75	
	AA	23	38	
rs17188118	AA	136	170	1.40 (0.67, 2.93)
	AC	11	19	
	CC	1	2	

DC: diabetic individuals without nephropathy, and DN: diabetic individuals with nephropathy.

**Table 4:** Results of association analysis between AKR1B1 variants and T2DM-nephropathy in healthy controls versus cases.

AKR1B1	Alleles	HT	DN	OR_G_
rs2259458	TT	43	28	0.94 (0.68, 1.30)
	TG	106	101	
	GG	90	63	
rs2734653	GG	138	102	1.10 (0.77, 1.57)
	GA	85	81	
	AA	16	7	
rs2670230	CC	88	51	1.24 (0.90, 1.71)
	CA	104	109	
	AA	47	33	
rs1790998	CC	93	80	0.94 (0.69, 1.30)
	CA	105	75	
	AA	45	38	
rs17188118	AA	206	170	0.95 (0.52, 1.73)
	AC	26	19	
	CC	1	2	

HT: healthy controls, and DN: diabetic individuals with nephropathy.

**Table 5:** Characteristics of studies included in meta-analysis.

Variant	Studies	Ethnicity	DM	N	Selection Criteria	N	Selection Criteria	N	Selection Criteria	Analyses
Genotype-based										
(CA)n (z > …. > z − 2)	Lajer (2004) [40]	Caucasians	T1DM	431	persistent macroalbuminuria, retinopathy	468	DM > 15 yrs and persistent normoalbuminuria	-	-	DC-C
	Park (2002) [41]	East Asians	T2DM	48	DM ≥ 10 yrs, macroalbuminuria and diabetic retinopathy	38	DM > 10 yrs with normoalbuminuria and without retinopathy	-	-	DC-C
	Liu (2002) [42]	East Asians	T2DM	137	persistent albuminuria	128	persistent normoalbuminuria	-	-	DC-C
	Heesom (1997) [43]	Caucasians	T1DM	75	persistent proteinuria without hematuria or infection and retinopathy	38	uncomplicated with DM ≥ 20 yrs	102	Healthy controls	DC-C, HT-DC-C, HT-C
	Moczulski (2000) [44]	Caucasians	T1DM	221	ESRD or persistent proteinuria	193	persistent normoalbuminuria	-	-	DC-C
	Wolford (2006) [45]	East Asians	T2DM	107	ESRD	108	DM ≥ 10 yrs, normoalbuminuria	-	-	DC-C
	Neamat-Allah (2001) [46]-North of England Cohort	Caucasians	T1DM	77	persistent proteinuria	85	DM ≥ 15 yrs, normoalbuminuria	67	Healthy controls	DC-C, HT-DC-C, HT-C
	Neamat-Allah (2001) [46]-North of England Cohort	Caucasians	T2DM	85	persistent proteinuria	148	DM ≥ 15 yrs, normoalbuminuria	67	Healthy controls	DC-C, HT-DC-C, HT-C
	Neamat-Allah (2001) [46]-Northern Ireland Cohort	Caucasians	T1DM	126	persistent proteinuria	116	DM > 20 yrs, normoalbuminuria	188	Healthy controls	DC-C, HT-DC-C, HT-C
	Neamat-Allah (2001) [46]- Pima Indians Cohort	East Asians	T2DM	182	persistent proteinuria	145	DM ≥ 10 yrs, normoalbuminuria	124	Healthy controls	DC-C, HT-DC-C, HT-C
−106 C>T (rs759853)	Neamat-Allah (2001) [46]-North of England Cohort	Caucasians	T1DM	77	persistent proteinuria	85	DM ≥ 15 yrs, normoalbuminuria	67	Healthy controls	DC-C, HT-DC-C, HT-C
	Neamat-Allah (2001) [46]-North of England Cohort	Caucasians	T2DM	85	persistent proteinuria	148	DM ≥ 15 yrs, normoalbuminuria	67	Healthy controls	DC-C, HT-DC-C, HT-C
	Neamat-Allah (2001) [46]-Northern Ireland Cohort	Caucasians	T1DM	126	persistent proteinuria	116	DM > 20 yrs, normoalbuminuria	188	Healthy controls	DC-C, HT-DC-C, HT-C
	Neamat-Allah (2001) [46]- Pima Indians Cohort	East Asians	T2DM	182	persistent proteinuria	145	DM ≥ 10 yrs, normoalbuminuria	124	Healthy controls	DC-C, HT-DC-C, HT-C
	Gosek (2005) [47]	Caucasians	T2DM	153	persistent microalbuminuria	162	DM ≥ 10 yrs, persistent normoalbuminuria	-	-	DC-C
	Moczulski (2000) [44]	Caucasians	T1DM	221	ESRD or persistent proteinuria	193	persistent normoalbuminuria	-	-	DC-C
	Sivenius (2004) [48]	Caucasians	T2DM	13	persistent albuminuria	-	-	126	Healthy controls	HT-C
	Prasad (2010) [49]	East Asians	T2DM	196	CRI, serum creatinine ≥ 3.0 mg/dL	225	DM ≥ 10 yrs, normal renal function and normoalbuminuria, matched for age and ethnicity	-	-	DC-C
	Dieter (2021) [50]	Brazilians	T2DM	695	persistent albuminuria	310	DM ≥ 10 yrs, persistent normoalbuminuria	-	-	DC-C
	Wolford (2006) [45]	East Asians	T2DM	107	persistent albuminuria and ESRD	108	DM > 10 yrs, persistent normoalbuminuria	-	-	DC-C
	Makiishi (2003) [51]	East Asians	T2DM	228	albuminuria	220	DM > 10 yrs, persistent normoalbuminuria			DC-C
Allele-based										
(CA)n (z > …. > z − 2)	Ichikawa (1999) [52]	East Asians	T2DM	41	persistent albuminuria	-	-	90	Healthy controls	HT-C
	Shah (1998) [53]-cohort UNMHSC	Mixed	T1DM	31	persistent albuminuria	27	persistent normoalbuminuria	33	Healthy controls	DC-C, HT-DC-C, HT-C
	Shah (1998) [53]-cohort HSR	Caucasians	T1DM	21	transplant of kidney and/or pancreas or persistent albuminuria	25	persistent normoalbuminuria	21	Healthy controls	DC-C, HT-DC-C, HT-C
	Dyer (1999) [54]	Caucasians	T1DM	211	persistent albuminuria, diabetic retinopathy without other kidney or urinary tract disease and hypertension	129	DM ≥ 15 yrs and persistent normoalbuminuria	-	-	DC-C
	Isermann (2000) [55]	Caucasians	T1DM	47	persistent albuminuria, retinopathy	42	DM > 20 yrs, persistent normoalbuminuria	-	-	DC-C
	Moczulski (1999) [56]	Caucasians	T2DM	295	persistent albuminuria	179	DM ≥ 10 yrs, persistent normoalbuminuria	-	-	DC-C
	Maeda (1999) [57]	East Asians	T2DM	216	persistent albuminuria	123	DM > 5 yrs, persistent normoalbuminuria	-	-	DC-C
	Ng (2001) [58]	Caucasians	T1DM	15	DM < 15 yrs, albuminuria and diabetic retinopathy	49	DM > 15 yrs, normoalbuminuria	42	Healthy controls	DC-C, HT-DC-C, HT-C
	Wang (2003) [59]	East Asians	T2DM	280	Persistent albuminuria	458	DM > 5 yrs, normoalbuminuria	-	-	DC-C
	Yamamoto (2003) [60]	East Asians	T1DM	68	Persistent albuminuria	134	persistent normoalbuminuria	-	-	DC-C
−106 C>T (rs759853)	Wang (2003) [59]	East Asians	T2DM	280	Persistent albuminuria	458	DM > 5 yrs, normoalbuminuria	-	-	DC-C
rs2361634, rs5918764, ss95212308	Tregouet (2008) [61]- Denmark Cohort	Caucasians	T1DM	489	Persistent albuminuria	463	DM > 15 yrs and persistent normoalbuminuria	-	-	DC-C
	Tregouet (2008) [61]- Finland Cohort	Caucasians	T1DM	387	Persistent albuminuria	469	DM > 15 yrs and persistent normoalbuminuria	-	-	DC-C
	Tregouet (2008) [61]- France Cohort	Caucasians	T1DM	300	Persistent albuminuria	391	DM > 15 yrs and persistent normoalbuminuria	-	-	DC-C

C: diabetic individuals with nephropathy, HT: healthy controls, DC: diabetic individuals without nephropathy, DM: diabetes mellitus, T1DM: Type 1 diabetes mellitus, and T2DM: Type 2 diabetes mellitus.

**Table 6:** Results from meta-analyses based on genotype counts.

Diseased Controls Versus Cases							
	Studies (n)	RE OR_G_	95% LL	95% UL	*I*^2^ (%)	*P_Q_*	*P_E_*
(CA)n (z > …. > z − 2)	10	1.162	0.962	1.404	60.015	0.007	0.491
*All in HWE*	2	1.397	0.992	1.968	28.278	0.238	-
*Caucasians*	6	1.035	0.857	1.251	45.136	0.105	0.753
*Asians*	4	1.455	1.042	2.032	52.523	0.100	0.717
*T1DM*	5	1.018	0.806	1.286	54.807	0.065	0.759
*T2DM*	5	1.357	1.039	1.771	50.628	0.088	0.479
−106 C>T (rs759853)	10	1.389	1.134	1.700	59.494	0.008	0.593
*All in HWE*	9	1.362	1.094	1.696	61.952	0.007	0.578
*Caucasians*	5	1.705	1.239	2.347	65.716	0.02	0.039
*Asians*	4	1.178	0.914	1.519	12.465	0.330	0.424
*T1DM*	3	2.046	1.431	2.927	46.968	0.152	0.131
*T2DM*	7	1.181	1.017	1.371	8.575	0.363	0.938
Healthy controls versus cases							
(CA)n (z > …. > z − 2)	5	0.991	0.847	1.159	0	0.789	0.232
*All in HWE*	1	1.172	0.689	1.995	-	-	-
*Caucasians*	4	0.968	0.804	1.164	0	0.686	0.206
*Asians*	1	1.053	0.782	1.418	-	-	-
*T1DM*	3	0.974	0.788	1.204	0	0.480	0.197
*T2DM*	2	1.011	0.801	1.277	0	0.667	-
−106 C>T (rs759853)	5	1.057	0.831	1.344	0	0.888	0.522
*All in HWE*	5	1.057	0.831	1.344	0	0.888	0.522
*Caucasians*	4	1.142	0.857	1.522	0	0.978	0.756
*Asians*	1	0.880	0.566	1.368	-	-	-
*T1DM*	2	1.139	0.798	1.627	0	0.810	-
*T2DM*	3	0.992	0.716	1.376	0	0.680	0.735
Healthy controls versus diseased controls versus cases							
(CA)n (z > …. > z − 2)	5	1.010	0.911	1.121	0	0.707	0.177
*All in HWE*	1	1.133	0.774	1.657	-	-	-
*Caucasians*	4	0.992	0.880	1.119	0	0.609	0.267
*Asians*	1	1.063	0.868	1.302	-	-	-
*T1DM*	3	0.998	0.865	1.152	0	0.406	0.305
*T2DM*	2	1.023	0.881	1.189	0	0.584	-
−106 C>T (rs759853)	4	1.078	0.922	1.260	0	0.842	0.310
*All in HWE*	4	1.078	0.922	1.260	0	0.842	0.310
*Caucasians*	3	1.121	0.935	1.344	0	0.934	0.275
*Asians*	1	0.963	0.709	1.310	-	-	-
*T1DM*	2	1.105	0.875	1.394	0	0.754	-
*T2DM*	2	1.056	0.856	1.304	0	0.419	-

**Table 7:** Results from meta-analysis based on allele counts between diseased controls versus cases.

	Studies (n)	RE OR	95% LL	95% UL	*P_E_*
(CA)n (z > …. > z − 2)	8	1.154	0.910	1.464	0.003
rs2361634	3	0.788	0.552	1.125	0.369
rs5918764	3	1.076	0.617	1.876	0.841
ss95212308	3	1.041	0.834	1.300	0.341

## Data Availability

The datasets used and/or analyzed during the current study are available from the corresponding author on reasonable request.
